# Online mentoring: challenges and strategies

**DOI:** 10.12669/pjms.38.8.5804

**Published:** 2022

**Authors:** Sadiq Jan, Usman Mahboob

**Affiliations:** 1 Dr. Sadiq Jan, FCPS. MHPE. Assistant Professor Obstetrics and Gynecology, Islamic International Medical College, Rawalpindi, Pakistan; 2 Dr. Usman Mahboob, MBBS, MPH, FHEA (UK), DHPE (UK), Fellow FAIMER (USA). Associate Professor Medical Education, Institute of Health Professions Education & Research, Khyber Medical University, Peshawar, Pakistan

**Keywords:** Mentoring, Online mentoring, E-mentoring, Mentors, Mentees, Challenges, Strategies

## Abstract

**Objectives:**

To gain insights into the e-mentoring experience, needs of the stakeholders (mentors and mentees) challenges and strategies to overcome the challenges.

**Methods:**

Qualitative exploratory study was conducted in Islamic International Medical College, from February 2021 to July 2021. The study duration was six months. Six ‘Semi-structured interviews’ of mentors and two ‘focus group discussions’ with mentees were conducted. A purposeful sampling technique was employed to select the respondents. Data were audio taped and transcribed verbatim. After that analysis of data was done by inductive content analysis. Data were coded line by line. Open codes were combined to form categories, which were combined to form themes through abstraction.

**Results:**

Data was analyzed by using Atlas.ti. After analyzing data from mentors and mentees, 21 open codes sorted into 15 categories and abstracted to from five major themes. Participants talked about the problems of online sessions like; connectivity issues, impaired interaction, nonspecific goals, unaware of MS Teams use. They suggested strategies to make these sessions more practical; like workshops for training, face to face sessions before online mode, blended approach, careful selection of mentors and mentees. All were satisfied with security and witnessed anonymity and privacy.

**Conclusion:**

Online mentoring can help students feel less lonely through social contact. E-mentoring provides flexibility to those who would usually deal with discrimination to being mentored because of their gender, ethnicity, disability or geographical location.

## INTRODUCTION

“Mentoring or Mentorship’’ has perhaps always existed back in history, like the relationship of Telemachus and the man responsible for his development, Mentor, who introduced his name to the notion first named in Homer’s epic, the Odyssey.^1^ Mentoring guides the mentees and the mentors to grow in academic activities, research and profession , therefore improving the outcome of patients, and improving the honor of the organizations involved.^2^,^3^ E-mentoring has the potential to develop a more effective relationship between mentors and mentees, therefore getting more consideration from academicians and industries. In the last decade online mentoring websites has grown phenomenally and have events of several professions. Online mentoring has emerged as a significant global trend. ‘Online mentoring is characterized by interaction and engagement via technology’.^4^

Online mentoring is defined^5^ “as the process in which electronic media are used as the main communication channel between the mentor and mentee. Coronavirus pandemic is an unprecedented global threat.^6^ During this pandemic traditional, on-campus students are challenged with campus closures and the inability to see their mentors face-to-face.^7^ These pressures typical to mentoring relationships are being more widely recognized. The foremost advantage of digital mentoring is that it is learner-centered.^8^ With technological advances and globalization of education, new innovative methods of online mentoring are available like emailing, videoconferencing, and document sharing.^9^ Given the recent increased need for online mentoring there is a vital need for research to gain insight into the online mentoring experience, the needs of the stakeholders’ (mentors and mentees) challenges and how these challenges can be overcome (strategies). Specific objectives of the study were to explore the challenges faced by mentees and mentors in online mentoring and to explore the views of mentors and mentees regarding strategies to resolve these challenges.

## METHODS

The study lasted six months, from February 2021 to July 2021. This study was conducted at “Islamic international Medical college, Rawalpindi”. IRB approval was taken from the Riphah University, (Appl. # Riphah /IRC/ 21/13 February 16^th^, 2021), which has already a well formed Mentoring program. This program was shifted from face to face to electronic mode during the pandemic. That’s why this study is planned to explore the new experience and address the challenges to suggest the strategies for improving the program. Non-probability purposive sampling was done. Students were contacted through class representatives and invited voluntarily to join.

Six interviews and two FGDs were conducted. During the data collection, the sufficiency of sample size was based on saturation of data, pragmatic considerations (literature-based), data richness and volume, research design requirements and sample size guidelines.^10^ In addition to FGDs, semi- structured interviews (SSI) using open-ended questions (produced from the literature) were employed to explore the views and experiences of the mentors and mentees. Inductive Qualitative content analysis of the data was done. It involves in depth analysis to identify the emerging themes from the data.^11^

Data of FGDs and SSI were analyzed separately. The whole transcript of each FGD was taken as a separate unit of analysis. One of the authors read the transcript multiple times, along with listening to the audios. Mistakes were corrected. Next, coding of the transcript was done line by line by highlighting and adding the comments. Then all codes were separated on another word document. All similar codes were combined to reduce the number. Similar codes were grouped into subcategories, combined to form generic categories, and lastly, generic categories were grouped under main categories/themes by abstraction.

The triangulation was conducted using two data sources: FGDs and semi-structured interviews. The participants were from different medical years and of both genders.

## RESULTS

Twelve students participated in two FGDs while six faculty members were interviewed as mentors, all from Islamic International Medical College Rawalpindi. Fifty percent mentees were girls and 50% were boys. Data were analyzed by using Atlas ti. After analyzing data 21 open codes emerged, sorted into 15 categories and abstracted to from five significant themes. Major themes which emerged were; Connection: a sense of community, goal orientation, security and control, digital support, technology related problems, and last one was regarding proposed strategies like; careful selection of participants, workshops for computer literacy, meet before e-mentoring and blended approach. details are mentioned in Table-I.

**Table-I:** Findings of the interviews and focus group discussions.

Ref	Main themes	Sub-themes/Categories	Codes	Frequency	Mentee quotations (FGDs)	Mentor quotation (Interviews)
1	Connection: A sense of community	A way of getting together	Adapting and developing the relationship	14	“I would say it’s pretty close to how it used to be in face-to-face mentoring.”	“… some of the students who weren’t very talkative during face-to-face sessions were much more comfortable in online sessions”
			Interaction	16		“Interaction is limited during online sessions,
		Balancing the nature of the relationship	Pairing of mentors and mentees	14	“….it is difficult to develop and balance the relationship”.	“….I do this by trying to see the situation from their perspective.”
		Relational trust:	Privacy	18	Yes ,we do discuss in private messaging.	“….we do discuss in private”.
2	Goal orientation	Mentees as goal oriented	Goal Orientation	18	“……their goals should be assigned separately.	“…… I motivate them and give them ideas, but they set their own goals.”
3	Security and control	Anonymity	Security	7	“We are pretty sure that our sessions remain confidential”	“it is very safe and secure.”
4	Digital support	Forum	Digital Support	10		“….we have Zoom, MS Teams and other options available. ”
		Messaging	Personal contact	14	“We have a WhatsApp group”.	
5	Technology related problems	IT equipment issues	Internet Problems	22	“We often have connectivity issues”	
		Computer literacy	Aware of computer use.	16	“i am well aware of computer use”.	“i know computer use”.
		Lack of facial expression	Cameras issue	31	“. The students don’t on their cameras.	
			Lack of Interaction			“The mentees stay quiet, there is no proper interaction”
		Cultural issues	Background variation	14	“‘Normally, the students with cultural difference do not normally talk”.	
6	Proposed strategies	Careful selection of participants	Switching Mentors		“Mentees should be allowed to switch their mentors.”	“We don’t have authority to choose who we will mentor.”
		Workshops for computer literacy	Technology related Problem solving	8		“training is needed for both the mentors and mentees.”
		Highly desirable to meet before e-mentoring	Unfamiliar with mentees			“Before online mentoring, there should be at least one physical interaction”.
		Blended approach	Comfort in face-to-face	04	“….intermittant face toface sessions along with online sessions, would be better”.	

**Table-II:** Action plan to improve online mentoring.

	Strategies	Action plans
1	Match making	• Have a mutual and trusted person facilitate the match.
		• Get an informal recommendation from a person who know both mentors and mentee.
2	Mutual commitment and follow through	• View interactions with each other as a commitment but not an obligation
		• Be open to foster the continued relationship in mentoring period.
		• Prioritize the commitments and follow through
		• Be responsive and timely in fulfilling the promises
3	Purposeful interactions	• Plan and be prepared for mentoring session
		• Use a tool like goals, deadlines to help track progress.
		• Be prepared ahead of time but be open for open discussions
4	Technology and in person meetings	• Schedule reoccurring meeting times.
		• Use teleconferencing technology with video rather than telephone only to foster connectedness and focused attention
		• Find opportunities to meet in person, like in conferences or invited visits

## DISCUSSION

Online mentoring, like any other innovation, is not free of critics. The much more significant potential audience can be benefited from the training of mentors, reasonable matching of mentors and mentees.^12^,^13^ These were the themes extracted from our data.

**1. *Connection: A sense of community:*** Mentorship will not always lead to establishing friendship but mutual respect and gratitude for the other person will help to foster an element of zeal to the experience that elevates the experience of mentoring.^14^ Participants from my study were well aware of the nature and frequency of interaction to develop an effective mentoring relationship and most of them are satisfied except very few. Literature also witnesses that interaction frequency is positively associated with e mentoring functions received.^15^ To improve the interaction, all stakeholders including mentors, mentees and the organization are working effectively in the said institute. This way they are adapting the new modality of online mentoring, including equipment, internet, multiple forums, alternate options, flexibility in timings etc.

***2. Goal orientation*** of mentors and mentees influences the mentoring program’s career-related mastery and effectiveness.^16^ In this study the mentees are also well aware of the importance of the goals. Mentors are also putting efforts at their end, but as it is already stated, online mentoring is a novice entity internationally and in our university; hence, it more training and robustness. Goal orientation is the most relevant construct in reducing the stress and anxiety in mentees.^17^ Students require establishing a positive relationship, and they feel supported by their mentors which is crucial to encourage dialogue and motivation throughout the learning process.^18^ Most of the mentors in study are putting effort to motivate the students. Training of mentors is needed to develop effective communication strategies for student’s motivation and confidence building.^19^

***3. Confidentiality and security*** are a critical components of an effective and successful mentoring relationship. It is thought to be doubtful in online modality.

***4. Digital support*,** few participants of study had reservations on Microsoft teams, which is one of the forums used in this institute. However, all were sure that the conversations and meetings are secure. A trustworthy relationship helps to open the discussions and allow progress towards the success.

***5. Information and communication technology (ICT) skills*** are interpersonal and exclusively one basis. Still, they are prone to interfere more than facilitating the formation and development of the online mentoring relationship. However, sometimes distance communication tools like online discussion, messaging, and video discussions, etc, supports and enhance the mentoring (coaching) relationship in educational settings.^20^ Participants use multiple methods and modalities to improve and help the mentoring relationship. Another component of mentoring is review of practice and performance can also be supported by technology like audio recording and videos.^20^ ICT resources can be used in training, development monitoring and supervision of mentees and mentors.^20^

E-mentoring being a new entity is not without critics. The major challenge of study participants mentioned the *lack of facial expressions* and the inability to read another person’s body language. Previous research also said this issue of online mentoring. One of the foremost critics of online mentoring is that it prevents mentor and mentee from observing the facial expressions and body language when it is asynchronous.^12^ But the advent of new technologies like online video mentoring has primarily accomplished this criticism. Another criticism is more difficult to overcome; participants may be less trusting of the mentoring relationship when it is online. Proposed strategies are about: *Confidentiality* of this relationship should be respected but online security may not be perfect. So there is the possibility that someone could find out details of the confidential mentoring relationship. Our participants have high level of confidence in each other and they don’t doubt in any breech of the trust. These issues of e-mentoring are the best approach to reduce their impact is that all participants must understand the risk involved and agree on the ground rules, as with face-to-face mentoring sessions.^12^ Few students who belonged to peripheral areas faced internet availability issues. Few mentors had reservations about using technology in the initial few sessions but they are in the improvement phase. Students are primarily aware of the use of technology. Internet-related challenges and level of *information technology proficiency* are the issues needed to be addressed. With time after training of mentors, *reasonable matching* of mentees and mentors, will make online mentoring programs meet the expectations of both sides as in face to face mentoring.^13^ While most of the mentees had suggestions that they should have the liberty to choose their mentors and should be allowed to change on the way during five years of medical life, whenever they need to switch to any other mentors. The common suggestion from most of the participants was *workshop training*, so that an adequate number of mentors can be trained at a time. Mentors had the opinion that responsive students were more accessible to mentor. In another study the researcher believed that students who “owned their process more and took the initiative and were more proactive in asking for support could stay on track and move through the process more quickly.^21^ Both the mentors and mentees believed that the mentor-mentee relationship could be better established if they get a chance to meet before starting online mentoring physically. The relationship of mentor and mentee may be reviewed and may have the provision of re-matching such groups.^21^ The mentors and the mentees both suggested that the mentoring take a *blended approach*, i.e. the mentoring to be online with intermittent physical sessions. *Multiple means of communication* can be used in the mentoring program, with the first face to face meeting; followed by a conversation on phone or skype, phone call, email, virtual classroom, Facebook etc. A blended communication model may be an ideal and realistic way for individuals to create and maintain their working and social relationship development in the weird world of current century.^22^

This study is of its kind that explored in-depth the perspective of mentors and mentees regarding online mentoring of medical students in Pakistan. This is the first study at the national level that addressed the challenges of online mentoring and proposed the strategies to improve it. We have suggested the action plan derived from our study’s qualitative data, which can help improve the online mentoring programs.

### Limitations of the study:

It was conducted in only one institute, if it had included multiple Centers, it could have derived more themes.

## CONCLUSION

E-mentoring provides flexibility to those who usually face hurdles to being mentored because of their gender, ethnicity, disability or geographical location. Mentees can take responsibility for initiating contact and participating actively in online discussions. Mentors also experience personal growth through mentoring, helping others in the profession, and providing advice and support. Online mentoring is an opportunity for innovation in mentorship. It’s still in a phase of evolution and can be made more effective if people’s concerns are addressed and challenges are resolved which needs a strategic planning according to the issues.

### Authors’ Contribution:

**SJ:** Conceived and designed the study. Data collection and manuscript writing and responsible for the integrity the manuscript.

**UM:** Editing and critical review of the manuscript.
